# Self- vs proxy-reported mobility using the mobility assessment tool-short form in elderly preoperative patients

**DOI:** 10.1186/s11556-018-0194-x

**Published:** 2018-04-26

**Authors:** Sunghye Kim, Michael E. Miller, Marina Lin, W. Jack Rejeski, Stephen B. Kritchevsky, Anthony P. Marsh, Leanne Groban

**Affiliations:** 1 Department of Internal Medicine, Section of General Internal Medicine, Wake Forest School of Medicine, Medical Center Boulevard, Winston-Salem, NC 27157 USA; 2 Sticht Center for Healthy Aging and Alzheimer’s Prevention, Wake Forest School of Medicine, Medical Center Boulevard, Winston-Salem, NC 27157 USA; 3 Division of Public Health Sciences, Department of Biostatistical Sciences, Wake Forest School of Medicine, Medical Center Boulevard, Winston-Salem, NC 27157 USA; 4 Department of Anesthesiology, Wake Forest School of Medicine, Medical Center Boulevard, Winston Salem, NC 27157-1009 USA; 5 Department of Health and Exercise Science, Wake Forest University, PO Box 7868, Winston Salem, NC 27109 USA; 6 Department of Internal Medicine, Section of Gerontology and Geriatric Medicine, Wake Forest School of Medicine, Medical Center Boulevard, Winston Salem, NC 27157 USA

**Keywords:** Mobility, Self-report, Proxy, Elderly

## Abstract

**Background:**

Mobility is fundamental to maintenance of an independent lifestyle and can predict clinical outcomes after health events among older individuals. However, certain clinical situations do not accommodate physical or self-assessments. This investigation examines whether proxy-reported assessments of function using the Mobility Assessment Tool-short (MAT-sf) form is a reliable alternative.

**Methods:**

Sixty-six older persons (≥ age 70) and their proxies were enrolled. Proxies rated patients’ mobility using the MAT-sf as did the patients.

**Results:**

The mean age of patients was 78.4 yr. (±6.2); 44% were female and 86% were white. Spouses made up 55% of the proxies, while 39% were children/in-laws. The correlation coefficient between patient and proxy MAT-sf scores was 0.81 (*p* < 0.01); a comparison of the slope of the regression line relating patient- and proxy-reported MAT-sf to a line of identity showed disagreement (*p* < 0.01), with proxy reports underreporting patient responses by 8.3% in lower mobility patients. The intra-class correlation characterizing agreement between repeated proxy reports 0.81.

**Conclusion:**

Proxy reports of mobility in older patients have good reliability. However, in patients with poor mobility, the proxies tend to report a lower mobility than the patients.

**Electronic supplementary material:**

The online version of this article (10.1186/s11556-018-0194-x) contains supplementary material, which is available to authorized users.

## Background

According to the U.S. Census Bureau projections, the number of persons age 65 years or older will increase from roughly 40 million in the year 2010 to 88.5 million in 2050; a net increase of 121%. During this same time period, the number of persons 85 years of age or older will increase by 227%; accounting for nearly 5% of the U.S. population. Both walking speed and physical function are predictors of mortality [1] and incident disability in this elderly population [2]. Mobility and functional status are also important predictors of outcomes after major health events such as hip fracture, acute myocardial infarction, and noncardiac surgery [1–4].

Traditionally, performance-based tests, such as timed up and go [5] and Short Physical Performance Battery (SPPB) [6] have been used both in research and clinical settings to assess physical function and mobility. However, the recent clinical utility [7] of a novel, video animated self-report measure of mobility – the Mobility Assessment Tool-short form (MAT-sf) [8] – has appeal since there are many times in healthcare settings that patients are unable or it is not feasible to conduct performance-based testing. The MAT-sf consists of 10 animated video clips of activities that span a wide range of functional capacity. The MAT-sf was validated against performance based test (SPPB, 400 m walk test) [7] and is known to predict surgical outcomes [9] and major mobility disability [10].

An interesting question that has been asked of those who developed the MAT-sf is whether or not the measure can be completed by a proxy. Indeed there are clinical situations such as critical illnesses, catastrophic trauma, or delirium where it is not possible for older adults to complete the MAT-sf on their own, yet it would be valuable for clinic staff to have information on patients’ level of functioning prior to their illness. Also, in addition to knowing whether proxy assessment of a significant other’s mobility can be used as a reliable surrogate of patients’ perceived mobility, it would be interesting to know whether there are systematic differences between patient and proxy perceptions of mobility, since this could be important in the design of treatment regimens.

### Aim of the study

The primary aim of this investigation was to examine the relationship between patients’ self-reported mobility and their proxies’ perceptions of the same using the MAT-sf. We also explored whether the relationship between patients’ and proxies’ assessments were stable across two different assessment points and whether level of function, and sex might be important in understanding these relationships.

## Methods

### Design and setting

This was a cross sectional study of 66 older patients and their proxies (e.g., age 70 and older) who presented to the Preoperative Assessment Clinic at Wake Forest Baptist Medical Center for elective surgical procedures. Patients who are scheduled for elective procedures visit the clinic for preoperative medical and anesthesiology assessment prior to the procedures. Since most of the patients visit the clinic with companions, we determined that this clinic is ideal place to enroll. Eligible patients and surrogates were asked to provide written informed consent (IRB approval number 00040417; approval date 11/29/2016) before undergoing standardized assessments for frailty and mobility status by trained study personnel.

### Inclusion criteria

We enrolled patients from January 17, 2017 to March 22, 2017, who met the criteria below: 1) age 70 years and older, 2) scheduled for elective surgical procedures, 3) accompanied by a proxy who was a caregiver of the patient or a family member and who spends a minimum of 1–2 h/day or 8 h/wk. with the patient. We enrolled an equal number of patients from lower, middle, and upper thirds of MAT-sf scores (lower ≤51.38 in men and ≤ 45.61 in women; middle 51.38 ≤ MAT-sf < 65.5 in men and 45.61 ≤ MAT-sf < 54.02 in women, and upper ≥65.5 in men and MAT-sf ≥ 54.02 in women). These subgroups were defined by our previous study of 197 elderly patients who were undergoing elective noncardiac surgeries [9].

### Exclusion criteria

We excluded patients or proxies who had a language barrier that would prohibit them from understanding the questionnaire and directions to use the MAT-sf. We also excluded patients or proxies who could not provide informed consent. Patients and proxies gave separate verbal informed consent. We excluded 36 patients including 21 patients who did not have proxies, 7 patients who refused to participate, 5 patients who were missed, and 3 patients who were wheelchair-bound. Enrolled patients and excluded patients did not statistically differ in age and gender.

### Mobility assessment

To assess mobility, participants were instructed on the use of the MAT-sf. The MAT-sf is a 10-item, computer-based assessment of mobility using animated video clips [11]. The 10 items used in the MAT-sf cover a broad range of functioning (walking on level ground, a slow jog, walking outdoors on uneven terrain, walking up a ramp with and without using a handrail, stepping over hurdles, ascending and descending stairs with and without the use of a handrail, and climbing stairs while carrying bags). Each item is accompanied by an animated video clip together with the responses for that question (number of minutes, number of times, yes/no). The tests were performed on a tablet (iPad, Apple, Inc) and were saved to an exportable file. While the patient is completing the MAT-sf assessment, the proxy was directed to a separate room to complete the MAT-sf for the patient (“Do you think Patient X can walk up this hill?”). To assess the stability of the proxy reported MAT-sf, the surrogates were asked to return within 2 weeks of the initial assessment and complete the MAT-sf on the patient a second time.

### Statistical analysis

We used descriptive statistics to characterize patients’ demographic variables, the relationship between the patients and their proxies, the time spent by the proxy with patients, and MAT-sf scores expressed as mean ± SD, median with interquartile range (IQR), or percentages as appropriate. We determined the mean ± SD of MAT-sf for both patients and proxies (both time points). Spearman correlations and regression analyses were used to quantify the association between patient and proxy responses. Interpretation of the correlation coefficient was exemplified by descriptive terms adapted from Munro [12] as follows: 0.00–0.25 little, if any, correlation; 0.26–0.49 low correlation; 0.50–0.69 moderate correlation; 0.70–0.89 high correlation; 0.9–1.00 very high correlation. The intraclass correlation coefficient was used to quantify intra-rater reliability assessments by the proxies [13]. Reliability measures greater than 0.75 have been previously categorized as representing excellent agreement [14]. Tests of the slope in the regression analyses, versus a slope of one, were used to quantify the refinement of proxy assessments relative to patient assessments. To assess the degree of agreement between patient and proxy response, the Bland-Altman analysis (including 95% confidence intervals) was also performed [15]. Mean differences between paired assessments of MAT-sf were performed using paired t-tests. All analyses were conducted using SAS 9.4 and *p* < 0.05 was considered statistically significant.

## Results

**Fig. 1:**
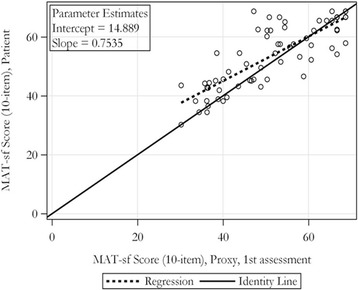
Regression estimates per MAT-sf score (self-reported vs proxy-reported)

**Fig. 2:**
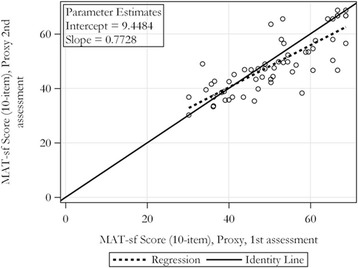
Regression estimates per MAT-sf score (first vs second proxy-reported)

We enrolled 66 patients and their proxies. The baseline characteristics of the patients and surrogates are summarized in Table 1. There were 24 patients in the lower MAT-sf tertile, and 21 patients in the middle and upper tertiles, respectively. The difference between patient and proxy-reported MAT-sf scores (proxy-reported MAT-sf minus patient reported MAT-sf) was − 1.3 (SD 5.4), − 0.43 (SD 7.8) and − 5.0 (SD 6.8) in the lower, middle and upper tertiles. There was significant agreement in patient-reported and proxy-reported MAT-sf scores (Table 2) with high correlation coefficients of 0.81 for proxy assessments with patients’ MAT-sf scores (*p* < .01). Test-retest reliability was also examined by comparing proxy-reported MAT-sf at first and second visits and the intra-class correlation was 0.81, exemplifying excellent agreement (*p* < .01). We plotted the patient versus the proxy reported MAT-sf at the initial time point to further investigate their congruency. The slope (beta = 0.76; SE = 0.76; *p* < .0001) was significantly different from the line of identity (r^2^ = 0.67, *p* = .0004 for test versus β = 1) with proxies reports at the lower end of MAT-sf (MAT-sf < 50; median of proxy reports = 51) reporting lower MAT-sf scores than the patients (8.3% lower; 4.3 absolute points; Fig. 1**)**. Neither age, sex, nor the type of proxy relationship (i.e., spouse, child, or in-law) explained a significant proportion of the variability in the difference between patients’ and proxies’ assessments after accounting for the proxy score. When the proxy reported MAT-sf at the first and second visit were plotted against one another, they also deviated from the line of identity (r^2^ = 0.67, *p* = .002) with the first assessment being, on average 6.4% higher, than the second assessment for initial MAT-sf scores ≥50 (Fig. 2). The Bland Altman plot conveys a similar account to that depicted in Figs. 1 and 2. Specifically, at the low end of MAT-sf scores (see Additional file 1: Figure S1A), the patient MAT-sf score has a tendency to be somewhat higher than the proxy score whereas at the high end, the proxy score is generally equal to or greater than the patient score. With respect to timing (see Additional file 1: Figure S1B), the first proxy assessment shows a tendency to be higher than the second proxy assessment at the high end of the MAT-sf score.

**Table 1:** Baseline characteristics of participants

Patients	
Age (years, SD)	78.4 (±6.2)
Female, n (%)	29 (43.9)
Race, n (%)	
Caucasian	57 (86)
African American	7 (11)
Other	2 (3)
Surrogates	
Relationship, n (%)	
Spouse	36 (55)
Child/In-law	26 (39)
Friend	2 (3)
Grandchild/In-law	2 (3)
Time spent with patients (per week)	24 h (IQR 8–24)
MAT-sf	
Patient reported (SD)	53.7 (10.8)
Proxy-reported (1st)	51.4 (11.8)
Proxy-reported (2nd)	49.2 (10.8)

**Table 2:** Patient and proxy reported MAT-sf (first, second visit) and Spearman correlation coefficient with *p* values (patient vs proxy)

	MAT-sf, patient	MAT-sf, proxy (1st)	MAT-sf, proxy (2nd)
MAT-sf, patient		Spearman = 0.81 (< .01) Mean Diff = 2.20 (SD = 6.87); *p* = .01 by paired t-test Percent Diff = 3.82 (12.4)	Spearman = 0.81 (< .01) Mean Diff = 4.30 (SD = 6.56); *p* < .0001 by paired t-test Percent Diff = 7.50 (11.71)
MAT-sf, proxy (1st)			ICC = 0.81 (< .01) Mean Diff = 2.25 (SD = 6.78); *p* = .01 by paired t-test Percent Diff = 3.24 (13.72)

## Discussion

We examined the agreement between self- and proxy-reported mobility using the MAT-sf in older patients and found that they were highly, and significantly associated, and these relationships were not moderated by age or gender. However, the association between proxies and patients significantly deviated from the line of identity indicating that proxy reports < 50 were lower than patients’ ratings. The test-retest reliability by proxy also showed excellent agreement, although the relationship deviated from the line of identity. The first assessment tended to be higher than the second assessment when proxies used higher MAT-sf scores.

Self-reported mobility assessment can overcome the constraints of time and space of performance based assessment and it is not limited by acute illness or symptoms to assess mobility [7]. This is especially helpful in clinical settings, since certain contexts or medical conditions make patient self-reporting either impractical or impossible. In situations where patients cannot provide necessary information, clinicians traditionally rely on patients’ proxies for data gathering (e.g., past medical history, medication list, health care decision) and these data are considered to be reliable. This study supports the position that the MAT-sf can also be collected by proxy, though the relationship between proxy and patients scores did deviate from the line of identity. With our findings, clinicians can confidently use the information on patients’ mobility that are assessed by a family member to predict the outcomes as well as to determine appropriate interventions in situations where patients cannot provide their mobility prior to the illnesses. What this discrepancy suggests is that proxies tend to rate patients’ mobility as lower than the patients themselves when proxies use scores < 50. The source of this difference is not known, although we believe that it may stem from the fact that proxies of patients with lower MAT-sf scores are more directly involved in providing support for mobility related activities of these patients, whether it is needed or not.

Although the reliability of proxy-reported mobility has never been reported, quality of life and impairments in cognitive function have been found to be lower when reported by proxy as compared to patients themselves. Specifically, in a study of 76 individuals who were ≥ 70 years of age with cognition ranging from no cognitive impairment to moderate dementia, the patient and proxy agreed on activities of daily living, but proxies reported lower quality of life related to executive function and greater impairment in the cognitive domain than patients, an effect that was deemed to indicate anosognosia of the patients [16]. Another study of community-dwelling Medicare beneficiaries of 76,115 person-years of patient-reports and 8822 person-years of proxy-reports demonstrated significantly higher percentages of health and functional limitations by proxy reports of patients than from patient reports [17]. Similar findings were demonstrated in adolescents: in a study of 323 adolescents (aged 15–18) and their parents, who enrolled in Florida’s Medicaid, parents reported lower pediatric Health Related Quality of Life (HRQoL) across all domains than did adolescents. When the difference was classified by the children’s BMI categories, the difference in physical functioning was greatest in obese adolescents [18]. In another study of 584 adolescents suffering from chronic conditions and their parents, the disagreement on the HRQoL between adolescents and their parents was relatively small but was affected by a few factors including number of hospital admissions (adolescent’s rating was higher (ADOL HIGH)), physical limitation (ADOL HIGH), and disease burden (ADOL HIGH). Likewise, it is possible that patients with poor functional status or mobility tend to overestimate their function and mobility compared to patients with normal or mildly impaired function or mobility.

Our study has a few limitations. First, this study was done at a single center, and the majority of the patients were white, compromising the generalizability of these findings. Second, the study did not have any performance-based test. Although we found that proxies reports at the lower end of MAT-sf reporting lower MAT-sf scores than the patients, this study cannot determine if the discrepancy is from the underestimation by the proxies or over-reporting by the patients. Also, differences in MAT-sf scores > 15 units, which are deemed to be statistically extreme based on Bland Altman plots (see Additional file 1), could have different implications at the two ends of the MAT-sf scale. To fully understand the clinical relevance of vast differences that might appear between patient and proxy scores at the ends of the MAT-sf spectrum, additional patient information might be necessary.

## Conclusion

In conclusion, we have shown that proxy reported mobility in older patients is reliable. However, in patients with poor mobility, the proxies tend to report their surrogates mobility lower than the patients themselves. A larger future study is warranted to fully appreciate the connection between patient and proxy across the mobility spectrum.

## Additional file

Additional file 1: **Figure S1.** Bland Altman comparison of MAT-sf scores between A) patient and proxy 1 and B) proxy1 and proxy 2. (PDF 71 kb)

## Abbreviations

IQR: Interquartile range
MAT-sf: Mobility Assessment Tool – short form
SPPB: Short Physical Performance Battery
