# Supplementary material for: Self- vs proxy-reported mobility using the mobility assessment tool-short form in elderly preoperative patients
Source: Eur Rev Aging Phys Act. 2018 Apr 26;15:5. doi: 10.1186/s11556-018-0194-x (PMC5918991; doi:10.1186/s11556-018-0194-x)
Supplement: Supplementary file 1 — Figure S1. Bland Altman comparison of MAT-sf scores between A) patient and proxy 1 and B) proxy1 and proxy 2. (PDF 71 kb) [file 11556_2018_194_MOESM1_ESM.pdf]

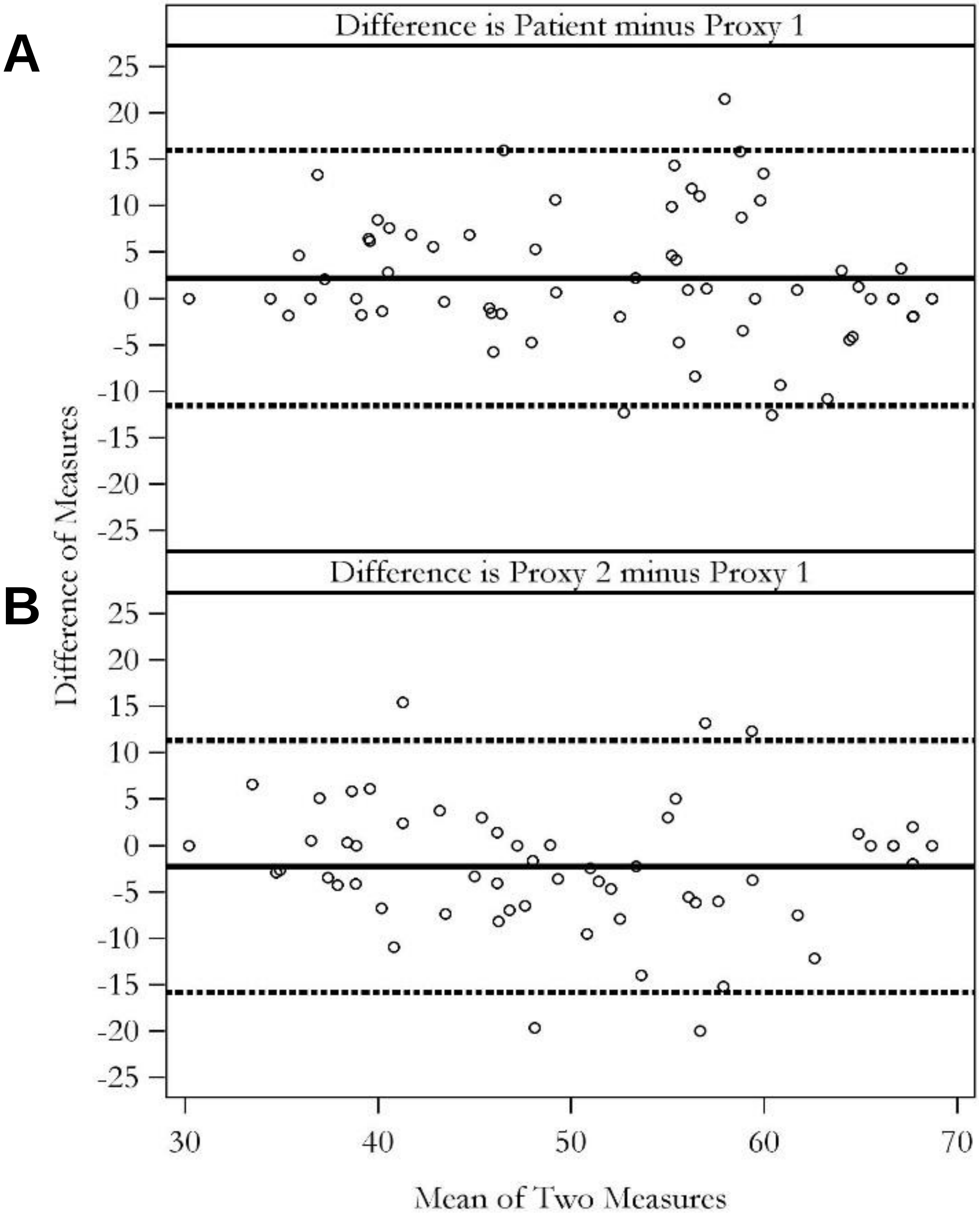

Supplemental Figure. Bland Altman comparison of MAT-sf scores between A) patient and proxy 1 and B) proxy1 and proxy 2
